# Ocular Side Effects of Dupilumab: A Comprehensive Overview of the Literature

**DOI:** 10.3390/jcm14072487

**Published:** 2025-04-05

**Authors:** Giacomo Boscia, Federico Spataro, Vanessa Desantis, Antonio Giovanni Solimando, Angelo Vacca, Roberto Ria, Alfonso Savastano

**Affiliations:** 1 Department of Translational Biomedicine Neuroscience, University of Bari “Aldo Moro”, 70121 Bari, Italy; 2 Guido Baccelli Unit of Internal Medicine, Department of Precision and Regenerative Medicine and Ionian Area (DiMePRe-J), University of Bari Aldo Moro, 70124 Bari, Italy; 3 Section of Pharmacology, Department of Precision and Regenerative Medicine and Ionian Area (DiMePRe-J), School of Medicine, University of Bari Aldo Moro, 70124 Bari, Italy; vanessa.desantis@uniba.it; 4 Guido Baccelli Unit of Internal Medicine, Department of Precision and Regenerative Medicine and Ionian Area (DiMePRe-J), School of Medicine, University of Bari Aldo Moro, 70124 Bari, Italy; antoniogiovannisolimando@gmail.com (A.G.S.); angelo.vacca@uniba.it (A.V.); roberto.ria@uniba.it (R.R.); 5 School of Medicine, Libera Università Mediterranea Degennaro, 70010 Casamassima, Italy; asavastano21@gmail.com; 6 Ospedale Generale Regionale F. Miulli, 70021 Acquaviva delle Fonti, Italy

**Keywords:** dupilumab, conjunctivitis, keratitis, blepharitis, ocular inflammation, dupilumab complications

## Abstract

Dupilumab, a monoclonal antibody targeting the interleukin (IL)-4 receptor alpha subunit and IL-13, has markedly advanced the treatment of atopic conditions such as dermatitis, asthma, and chronic rhinosinusitis. However, its expanding use has brought increased attention to a range of ocular adverse events—conjunctivitis, blepharitis, keratitis, corneal ulcers, and cicatricial conjunctivitis—that remain underrecognized and frequently underestimated in clinical practice. These manifestations often emerge in patients with atopic dermatitis and display varying severity, posing diagnostic and therapeutic challenges. Rather than isolated phenomena, these effects appear to stem from a complex interplay of goblet cell depletion, mucin deficiency, immune dysregulation, and microbiome alterations, including Demodex proliferation. Current management strategies remain largely empirical, lacking standardized protocols, and are often guided by anecdotal evidence. In this review, we critically appraise the existing literature, synthesize emerging pathogenic hypotheses, and highlight the unmet clinical need for evidence-based treatment algorithms. We advocate for a multidisciplinary approach and future research aimed at elucidating mechanisms, refining risk stratification, and minimizing ocular toxicity without compromising the therapeutic benefits of dupilumab. Furthermore, we intend to provide a more practical and straightforward resource for the reader based on the current literature on approaching the topic.

## 1. Introduction

Dupilumab is the first biologic therapy approved by the U.S. Food and Drug Administration (FDA) and the European Medicines Agency (EMA) for treating a variety of atopic conditions, including atopic dermatitis, asthma, and chronic rhinosinusitis with nasal polyps [1]. Dupilumab is a human monoclonal antibody, targeting the interleukin (IL)-4α, a subunit of interleukins IL-4, and IL-13, which are pivotal in driving the inflammatory processes behind these diseases [2]. Clinical trials have demonstrated its remarkable efficacy in managing moderate-to-severe atopic dermatitis, leading to significant improvements in the Eczema Area and Severity Index (EASI), itching, and overall quality of life [3]. Furthermore, dupilumab has proven effective in reducing asthma exacerbations, improving lung function, and alleviating symptoms of chronic rhinosinusitis with nasal polyps [4,5].

However, despite its therapeutic benefits, dupilumab has been linked to several side effects ranging from nasopharyngitis and headache to psoriasiform manifestations and increased eosinophil levels [6]. Ocular side effects are among the most common side effects of dupilumab [5]. The ocular side effects of dupilumab can vary from mild forms, such as conjunctivitis, to less frequent but more severe conditions, such as cicatrizing conjunctivitis [5,7,8,9]. The most common dupilumab-related complication remains conjunctivitis, frequently occurring in patients undergoing therapy for atopic dermatitis [10]. These forms are usually mild and rarely necessitate the drug’s discontinuation [1].

Akinalde and colleagues, by examining clinical trials of patients treated with dupilumab for various conditions, provided a broader perspective on the incidence and risk factors associated with the development of complications [10]. Interestingly, clinical trials reporting dupilumab use for other disorders than atopic dermatitis described a lower rate of conjunctivitis [10,11,12,13]. Factors such as the initial severity of atopic dermatitis, a prior history of conjunctivitis, and elevated baseline levels of eosinophils and IgE have been correlated with an increased risk of developing conjunctivitis [10]. Notably, patients who respond fully to dupilumab tend to experience fewer ocular complications [10]. Nevertheless, the mechanisms and predisposing factors underlying dupilumab-related ocular complications have not yet been fully clarified.

As dupilumab use continues to expand across multiple atopic diseases, vigilant monitoring and management of these ocular effects are crucial. Consequently, there is an urgent need for a standardized approach to diagnosing and managing ocular complications associated with dupilumab. This review aims to offer a comprehensive overview of the current literature about these ocular manifestations, providing insights into their pathogenesis, with a focus on related management.

## 2. Methods and Literature Search

In the present study, we performed a comprehensive search on the Web of Science, Pubmed, EMBASE, and ScienceDirect databases, using the following terms: “ocular side effects”, “dupilumab”, “ocular complications”, “conjunctivitis”, “cicatricial conjunctivitis”, “keratitis”, “punctal stenosis”, “blepharitis”, “periocular dermatitis”, and “management of dupilumab side effects”. We included articles up to January 2025. References from relevant studies were also examined to ensure comprehensive coverage of the topic. We limited our research to studies written in English language or to those written in other languages but with an English abstract if the abstract reported adequate information. Articles without English abstracts were excluded. Case reports were included only if they provided relevant or innovative information.

## 3. Etiopathogenic Mechanisms

The article thoroughly explores the mechanisms that underlie dupilumab-induced ocular side effects. By inhibiting the IL-4 and IL-13 signaling pathways, dupilumab alters immune responses that are crucial for maintaining the homeostasis of the ocular surface. Specifically, a blockade of these immune pathways has significant repercussions on the ocular surface, leading to dupilumab-associated conjunctivitis (DAC) and dupilumab-associated mucin deficiency (DAMD). One of the primary mechanisms underlying these adverse effects is the reduction of conjunctival goblet cells and mucin (MUC5AC) production. IL-13 plays a pivotal role in goblet cell proliferation and maintenance, as well as in the synthesis of MUC5AC, the primary mucin responsible for tear film stability. By blocking IL-13, dupilumab drastically decreases goblet cell density, resulting in mucin deficiency, tear film instability, ocular surface dryness, and epithelial barrier dysfunction. Histopathological analyses of conjunctival biopsies from dupilumab-treated patients reveal a significantly reduced goblet cell density (3.3 cells/mm^2^ vs. 32.3 cells/mm^2^ in controls), accompanied by a prominent inflammatory infiltration of CD3+/CD4+ T cells and eosinophils [14]. Beyond goblet cell depletion, an IL-4/IL-13 blockade may alter the local immune balance, favoring the activation of alternative inflammatory pathways. Notably, the lack of IL-4/IL-13 signaling may lead to increased OX40L pathway activity, thereby exacerbating pre-existing allergic conjunctivitis. OX40, a co-stimulatory receptor expressed in activated T cells, interacts with its ligand OX40L, found in antigen-presenting cells, to prolong T cell survival and enhance inflammatory responses [15]. Under normal conditions, IL-4 and IL-13 help regulate immune homeostasis, but their inhibition by dupilumab may lead to a compensatory upregulation of OX40/OX40L signaling, resulting in prolonged T cell activation and persistent conjunctival inflammation [15]. Additionally, a transient increase in circulating eosinophils following dupilumab initiation has been observed, potentially contributing to ocular inflammation via the release of cytotoxic inflammatory mediators that damage the conjunctival epithelium. Another proposed mechanism involves conjunctival dendritic cells. Goblet cells not only secrete mucins but also interact with dendritic cells to maintain an immunotolerant environment on the ocular surface. Goblet cell loss may disrupt this homeostasis, leading to chronic inflammation and immune dysregulation.

**Figure 1:**
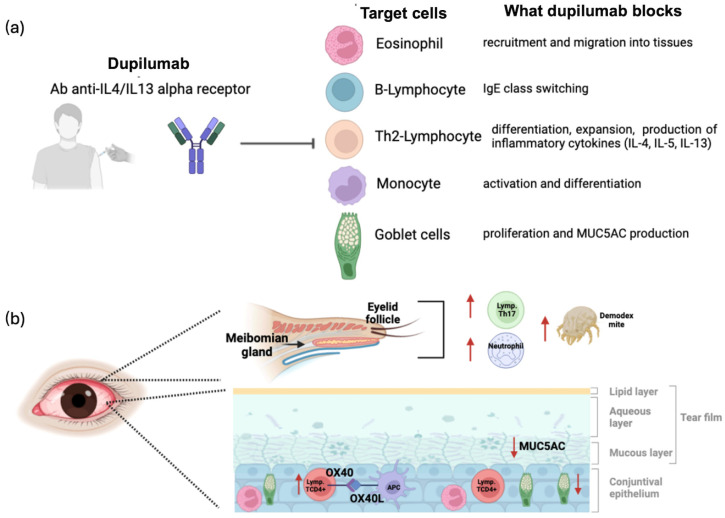
Mechanism of ocular side effects induced by dupilumab. (**a**) Dupilumab, a monoclonal antibody against the IL-4/IL-13 receptor alpha, is administered subcutaneously. It reduces the function of various immune system cells, including eosinophils, Th2 and B lymphocytes, monocytes, and mucin-producing cells associated with the airway mucosa. (**b**) The inflammatory pathway leading to blepharitis (**above**) and conjunctivitis (**below**) is shown. Red upward arrows indicate an increase, while downward arrows indicate a reduction. Lymp, lymphocyte.

Another emerging factor in the pathogenesis of DAC is the potential role of *Demodex* mite infestation, which has been hypothesized to increase following dupilumab therapy. *Demodex folliculorum* and *Demodex brevis* are commensal mites that colonize the hair follicles and sebaceous glands of the eyelids, typically in low numbers without causing significant pathology [16]. However, an overgrowth of *Demodex* can lead to chronic blepharitis, meibomian gland dysfunction, and ocular surface inflammation. IL-4 and IL-13 play an important role in regulating skin and mucosal immunity, including mechanisms that help control *Demodex* populations. By inhibiting these cytokines, dupilumab may inadvertently create a permissive environment for *Demodex* proliferation, exacerbating periocular inflammation and contributing to ocular surface disease. This altered ocular microenvironment could explain why some dupilumab-treated patients develop a rosacea-like ocular condition, distinct from classic allergic conjunctivitis. Unlike the Th2-driven inflammation typically seen in AD, *Demodex*-associated ocular rosacea is more Th17-driven, characterized by a predominance of CD4+ T cells, neutrophils, and chronic epithelial irritation rather than eosinophilic inflammation [16]. Clinically, this condition manifests as persistent eyelid inflammation, telangiectasia, and meibomian gland dysfunction, often resistant to standard anti-inflammatory therapies (Figure 1).

These pathophysiological alterations are further supported by clinical findings. A study by Achten et al. [17] demonstrated that 28.9% of dupilumab-treated AD patients developed DAC, with a decrease in the percentage of MUC5AC-producing goblet cells, despite a stable goblet cell count. Notably, 90% of patients already exhibited signs of ocular surface disease before starting dupilumab, suggesting that pre-existing ocular inflammation may predispose DAC [17].

In summary, the key etiopathogenetic mechanisms of dupilumab-associated ocular side effects include goblet cell depletion, MUC5AC deficiency, tear film instability, T cell and eosinophilic inflammation, loss of immune tolerance in the conjunctiva, and potential alterations in the periocular microbiome. These combined mechanisms explain the high prevalence of DAC and DAMD in AD patients undergoing dupilumab therapy, emphasizing the need for targeted ophthalmologic management strategies to mitigate these ocular complications.

## 4. Complications and Management Strategies

The most commonly reported complications include conjunctivitis, blepharitis, and keratitis. Among the most severe complications, cicatricial conjunctivitis and corneal ulcer may also occur. Herein, each complication is addressed individually, with management strategies outlined for each.

### 4.1. Conjunctivitis

Conjunctivitis is the most frequently reported ocular adverse event associated with dupilumab, particularly in patients treated for atopic dermatitis [1,10]. The incidence of conjunctivitis in clinical trials has been reported to range from approximately 4.7% to 22.1% [10,18]. Akinalde and colleagues provided an overview of dupilumab-associated conjunctivitis by analyzing six randomized, placebo-controlled clinical trials evaluating the efficacy of dupilumab for various conditions, including atopic dermatitis, asthma, chronic rhinosinusitis with nasal polyps, and eosinophilic esophagitis [10]. Their findings indicated that patients with atopic dermatitis treated with dupilumab had a higher incidence of conjunctivitis compared to those receiving a placebo [10]. In contrast, they found that dupilumab treatment was not associated with an increased incidence of conjunctivitis in patients with other conditions [10]. Additionally, they identified atopic dermatitis, a prior history of conjunctivitis, and elevated baseline levels of eosinophils and IgE as risk factors for developing conjunctivitis [10].

Although the exact reason for this difference in incidence remains unclear, the authors speculated that epithelial barrier dysfunction, observed in patients with atopic dermatitis and affecting both the cutaneous and ocular surface epithelia, could partially explain the higher prevalence of conjunctivitis in this population [10,19]. However, across all included trials, most cases of conjunctivitis were mild to moderate and were resolved without requiring discontinuation of dupilumab [10]. Symptoms typically appear around two weeks after the initiation of dupilumab monotherapy and between four and eight weeks after starting combination therapy with corticosteroids [10].

**Figure 2:**
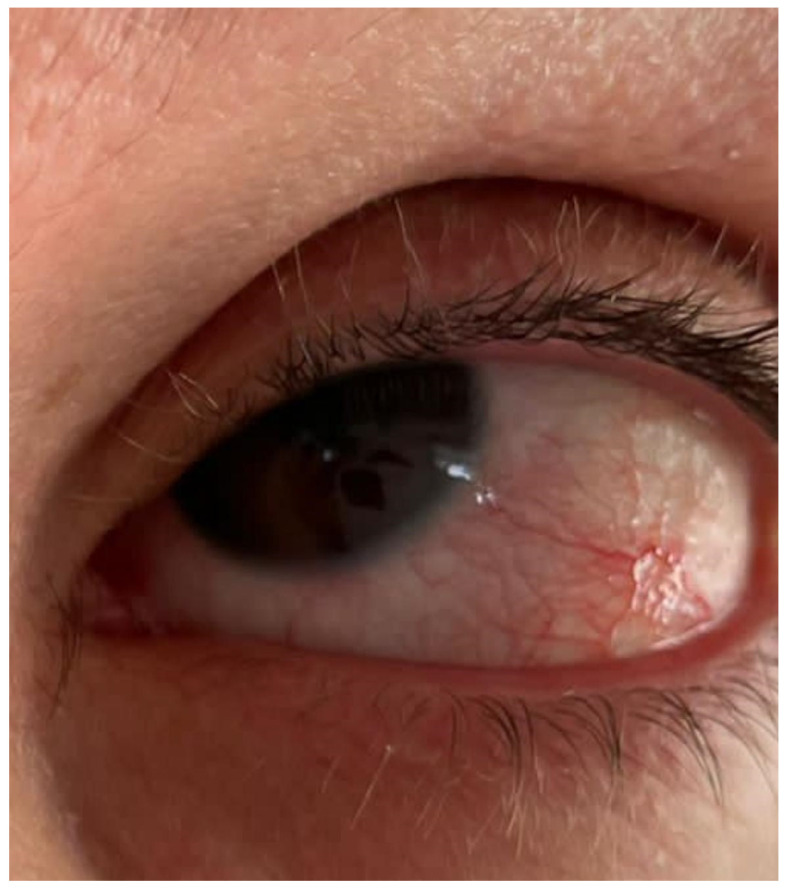
A case of dupilumab-associated conjunctivitis.

Dupilumab-associated conjunctivitis presents with a range of clinical signs varying in severity. Common findings include conjunctival hyperemia, chemosis, and papillary, follicular, or filamentous conjunctival reactions [7,20,21,22,23,24,25,26], often accompanied by ocular pruritus, tearing, and mucous discharge [5]. In more severe cases, conjunctivitis may be associated with punctate epithelial erosions, meibomian gland dysfunction, and tear film instability, exacerbating symptoms of ocular dryness and irritation [5]. Slit lamp examination may reveal inflammatory cell infiltration and goblet cell loss, further contributing to ocular surface dysfunction [14,27]. While most cases are mild to moderate and self-limiting, some patients may develop chronic or recurrent conjunctivitis, necessitating ongoing management with topical corticosteroid agents and regular ophthalmologic monitoring [10]. Cicatricial conjunctivitis will be discussed in the paragraph below (Figure 2).

#### Management

Management of dupilumab-associated conjunctivitis involves a stepwise approach depending on severity. Wu and colleagues proposed a management algorithm for dupilumab-associated conjunctivitis and other complications [5]. Mild-to-moderate cases often improve with preservative-free artificial tears, trehalose, or sodium hyaluronate, cold compresses to reduce irritation, and topical antihistamines (e.g., olopatadine) or mast cell stabilizers, which can help control inflammation [5,22,23,28,29]. More severe cases may require short courses of topical corticosteroids (e.g., loteprednol or fluorometholone) or calcineurin inhibitors (e.g., tacrolimus 0.03% ointment or cyclosporine 0.05% drops) [5,30]. In 2019, Bakker and coworkers reported the treatment of six cases of dupilumab-associated conjunctivitis with ocular anti-inflammatory treatment [14]. Shen and associates reported the improvement of two cases of follicular conjunctivitis after treatment with prednisolone acetate, and with olopatadine and cyclosporine, respectively [24]. Subsequently, Wollenberg and associates reported the treatment of 11 cases of dupilumab-associated conjunctivitis [31]. Specifically, two patients with mild symptoms underwent a treatment with hyaluronic eye drops, while another five cases were treated with fluorometholone 0.1% eye drops. The rationale of the latter treatment, previously reported in dupilumab-associated conjunctivitis [32], is inducing a reduction of the allergic inflammation, while having low penetration in the anterior chamber [31]. All five patients showed significant improvement of the symptoms [31]. Three patients, presenting more severe conditions, underwent topical treatment with steroids, reporting significant improvements [31]. Furthermore, the authors described also the approach with tacrolimus 0.03% eye ointment for the treatment of dupilumab-associated conjunctivitis, in order to allow a long-term treatment without steroids-induced complications [31]. Alternatively, for severe follicular conjunctivitis, treatment with cyclosporine eye drops may be considered [22,31]. Other authors reported the treatment of moderate-to-severe cases with oral doxycycline and oxytetracycline [30,33,34]. The approach with tetracyclines is already widely used for the treatment of ocular surface inflammation due to their bacteriostatic anti-inflammatory action [35,36]. In refractory cases, discontinuation of dupilumab may be considered, though this is typically reserved for sight-threatening complications [5,22,30]. Deleuran and coworkers, in a randomized controlled trial including 1491 patients treated for atopic dermatitis, reported 160 (10.7%) conjunctivitis episodes of various severity [37]. Among them, three patients (0.2% of total) discontinued dupilumab because of conjunctivitis-related adverse effects [37]. In any case, after the onset of the episode, regular ophthalmologic check-ups are recommended to monitor its progression.

### 4.2. Blepharitis

Dupilumab has also been implicated in cases of blepharitis. As for the other dupilumab-induced ocular surface disorders, it is thought that dupilumab’s inhibition of IL-4 and IL-13 signaling leads to an inflammatory imbalance that may contribute to tear film instability, meibomian gland dysfunction, goblet cell deficiency, and blepharitis [1]. The exact incidence of dupilumab-induced blepharitis is unclear due to its absence in clinical trial reports [5]. In a prospective single-center study, Touhouche and coworkers found that 31.3% of patients developed new-onset blepharitis after starting dupilumab for atopic dermatitis treatment, despite having no prior history [38]. Retrospective reports also document cases, though without baseline ophthalmologic exams, making causality uncertain [5,8,25,39]. Given that atopic dermatitis patients are 11 times more likely to develop blepharitis and are the primary recipients of dupilumab [40], the condition may be underreported [5]. While an exact incidence is unknown, the available data suggest that blepharitis could be a frequent side effect of dupilumab, particularly in atopic dermatitis patients [5].

**Figure 3:**
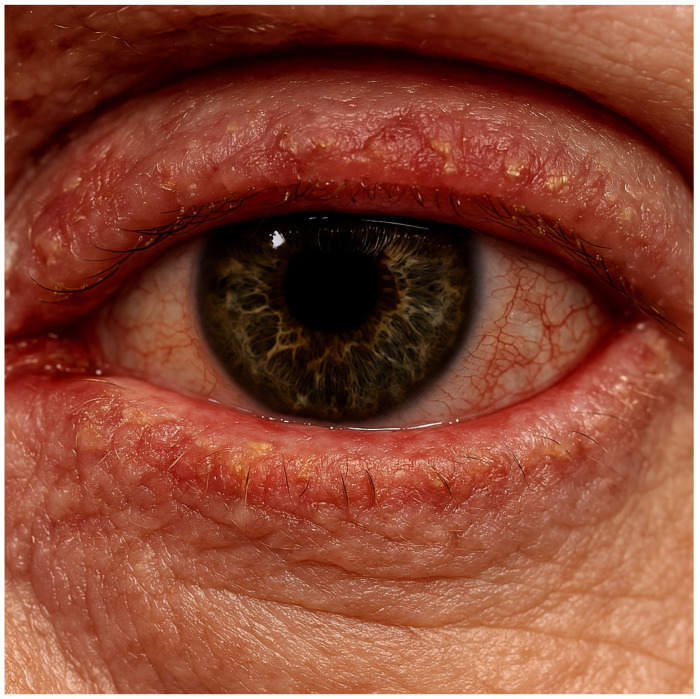
A case of dupilumab-associated blepharitis.

Patients often present with eyelid redness, irritation, foreign body sensation, burning, itching, discomfort, dry eyes, and tearing. Blepharitis may appear in three different forms: anterior, posterior (meibomitis), or a combination of both the previous forms [5]. Anterior blepharitis primarily affects the base of the eyelashes and the outer edges of the eyelids, often causing redness, scaling, crusting, and irritation [41,42]. This type is commonly associated with bacterial overgrowth or Demodex mite infestation [43]. On the other hand, posterior blepharitis, also known as meibomitis, involves inflammation of the meibomian glands within the eyelids, leading to gland dysfunction, irregular oil secretion, and tear film instability [41,42]. Patients with this form frequently report dry eyes, a burning sensation, and eyelid swelling [42]. In many cases, both anterior and posterior blepharitis develop simultaneously, resulting in a mixed presentation with overlapping symptoms [42]. Regardless of the specific type, dupilumab-induced blepharitis can lead to significant ocular discomfort and often necessitates targeted treatment strategies to control inflammation and maintain proper eyelid hygiene [41] (Figure 3).

#### Management

Wu and colleagues also proposed a management algorithm for blepharitis [5]. The mainstay of treatment for dupilumab-induced blepharitis includes meticulous eyelid hygiene with warm compresses and gentle lid scrubs using diluted baby shampoo or commercial lid cleansers [5]. Artificial tears can provide symptomatic relief for associated dryness. In moderate-to-severe cases, topical corticosteroids (e.g., loteprednol) or calcineurin inhibitors (e.g., tacrolimus) can reduce inflammation [5]. Oral doxycycline or azithromycin may be beneficial in refractory cases due to their anti-inflammatory and meibomian gland-modulating effects [5].

Moreover, it is important to consider the possible presence of Demodex in anterior blepharitis. In such cases, the use of a tea tree oil-based eyelid scrub is recommended, as it can help reduce the infestation and improve symptoms [5].

### 4.3. Keratitis and Corneal Ulcers

Dupilumab-induced ocular surface inflammation, tear film instability, and goblet cell dysfunction may also cause keratitis [1]. We have several reports in the literature of dupilumab-induced keratitis [23,25,30,39,44,45,46]. Clinically, the majority of articles reported the presence of a superficial punctate keratitis [23,25,30,38,44,45,46], while corneal ulceration was reported only in more severe cases, [47]. Slit lamp examination may reveal corneal staining with fluorescein [22], reduced tear break-up time [23], and conjunctivitis [23]. Maudinet and associates described in six patients undergoing a therapy with dupilumab, mild conjunctivitis associated with inferior punctuated corneal epithelial lesion [22]. All cases presented with a TBUT (Tear Film Beak up Time) of less than 10 s [22]. A similar case was reported by Paulose and colleagues [23]. In such cases, superficial punctate keratitis was associated with a significant papillary conjunctivitis, bilateral meibomian gland dysfunction, edematous eyelids with multiple chalazia [23]. Patients experienced ocular discomfort, redness, photophobia, blurred vision, foreign body sensation, increased tearing, and sensitivity to light, which can significantly impact daily activities [23,25,44,45,46]. A more severe case was reported by Li and coworkers [48]. Indeed, they described a case of a patient presenting a dupilumab-induced unilateral inferior, paralimbal sterile corneal ulceration in his right eye [47]. Conversely, no active keratitis was reported in the left eye [47].

#### Management

The management of keratitis depends on the severity of the case [5]. Mild-to-moderate cases often improve with preservative-free artificial tears, warm compresses, trehalose or sodium hyaluronate, and topical antihistamines (e.g., olopatadine or ketotifen), or mast cell stabilizers, which can help control inflammation [5,22,30]. Other authors described the dexamethasone application alone or in combination with artificial tears [20,25]. Severe keratitis or vision-threatening cases, such as corneal ulceration, have been managed with the temporary discontinuation of dupilumab and the initiation of antibiotic drops like moxifloxacin [47].

### 4.4. Punctal Stenosis

Punctal stenosis, or the narrowing of the lacrimal puncta, has been observed as a rare but potentially significant adverse effect of dupilumab therapy [4]. Chronic ocular surface inflammation induced by dupilumab may lead to fibrotic changes in the punctal region, impairing tear drainage and exacerbating dry eye symptoms [4]. Patients present with epiphora and ocular discomfort [4]. Levine and coworkers reported a case of a patient in treatment with dupilumab for atopic dermatitis, presenting with cicatrizing blepharoconjunctivitis and punctal stenosis [8]. Lee and associates presented three cases of punctal stenosis and conjunctivitis in patients undergoing a treatment with dupilumab [7]. Among them, one case was progressed to punctal obstruction [7]. In both reports, all the cases of punctal stenosis were found to be associated with conjunctivitis [7]. Thus, Lee and colleagues hypothesized the potential role of conjunctivitis-related inflammation in punctal stenosis development [7].

#### Management

For punctal stenosis treatment, Levine and coworkers, in agreement with the patient’s preferences, reduced the dupilumab administration frequency and started a treatment with dexamethasone 0.1% eyedrops [8]. After one month of ocular treatment, the symptoms gradually improved, and after six months, were near completely resolved [8]. In a report by Lee et al., the first case resolved after dupilumab discontinuation, while the other cases refused the treatment interruption and underwent a topical treatment with erythromycin, prednisolone acetate, loteprednol, and artificial tears [7]. In that case, symptoms progressed to punctal obstruction, and clinicians performed a surgical intervention with probing, punctoplasty, and silicone intubation [7].

### 4.5. Cicatricial Conjunctivitis

Dupilumab-induced cicatricial conjunctivitis is a rare but significant adverse effect of dupilumab [5]. Cicatricial conjunctivitis is a chronic inflammatory condition of the conjunctiva that leads to progressive scarring and fibrosis [32]. It can result from various causes, including autoimmune diseases, infections, drug reactions, and chemical or thermal injuries [48]. Characteristic symptoms include persistent ocular redness, irritation, dryness, foreign body sensation, photophobia, and blurred vision [5,7,8,32,49]. Levine and colleagues described a cicatrizing blepharoconjunctivitis presenting with subepithelial fibrosis [8]. Clinical presentation includes different levels of conjunctival hyperemia and inflammation [5]. Additional complications are symblepharon formation, forniceal shortening, and ectropion [5,7,8,32]. In 2017, Barnes and associates described the first case of a patient with dupilumab-induced conjunctivitis associated to cicatricial ectropion [32]. Subsequently, a similar case was reported by Lee and colleagues [7]. They described a case of dupilumab-induced conjunctivitis associated to cicatricial ectropion, papillae, secondary lagophthalmos, and punctal stenosis [7]. Nettis et al. described similar findings presenting bilaterally in a patient with a long-standing history of dupilumab administration for atopic dermatitis [50].

#### Management

Dupilumab-induced cicatricial conjunctivitis is a severe adverse effect of dupilumab requiring an early diagnosis and prompt intervention. Discontinuation of dupilumab was considered in all the cases previously reported [7,8,32,49]. Oral prednisolone administration to control inflammation was also prescribed [32,49]. As a topical treatment, the use of 0.1% dexamethasone eyedrops and topical cyclosporine has been described [8,49]. Interestingly, in the article published by Levine and coworkers, cicatricial conjunctivitis resolution was achieved with dupilumab reduction, but not discontinuation, and with dexamethasone 0.1% eyedrops administration [8].

### 4.6. Periocular Dermatitis

Dupilumab-induced periocular dermatitis is a recognized adverse effect of dupilumab therapy, primarily affecting patients treated for atopic dermatitis [4]. It typically presents with erythema, scaling, and edema around the eyes, often resembling eczematous dermatitis [7,22,46,50]. Furthermore, such conditions are often accompanied by conjunctivitis, meibomian gland dysfunction, or blepharitis, further exacerbating ocular discomfort [7,22,46,50]. Yamane and coworkers presented two cases of dupilumab-related periocular dermatitis [46]. Given the specific location in periocular skin, they hypothesized that the reduced skin thickness around the eyelids somehow facilitated the passage of the antigen, consequently leading to periocular irritation [46]. Lee and associates reported the association of periocular dermatitis, punctal stenosis, and conjunctivitis in patients undergoing a treatment with dupilumab for a severe form of atopic dermatitis [7]. A similar case was also reported by Maudinet and associates [22]. Clinical symptoms include burning and irritation, as well as other signs commonly linked to ocular inflammation [46].

#### Management

In these cases, topical treatment included preservative-free artificial tears, trehalose, or sodium hyaluronate, associated with dexamethasone or fluorometholone [22,46]. Maudinet and associates described a complete resolution after topical treatment with trehalose and dexamethasone [22]. Discontinuation of dupilumab was also considered in more severe cases [7,46]. In a report by Lee et al., while one patient discontinued dupilumab, the other two cases refused the drug interruption, undergoing a topical treatment with erythromycin, prednisolone acetate, loteprednol, and artificial tears [7]. Yamane and coworkers prescribed dupilumab discontinuation in one case, and a therapy with oral methylprednisolone and cetirizine in addition to topical periocular tacrolimus and hydrocortisone cream in the second one, obtaining a complete resolution in both patients [46].

### 4.7. Additional Miscellaneous Complications

Padidam and associates reported a case series of four instances of uveitis following dupilumab treatment for atopic dermatitis [9]. Each patient developed distinct manifestations, including posterior scleritis, anterior and intermediate uveitis with cystoid macular edema, relentless placoid chorioretinitis, and bilateral cystoid macular edema [9]. Symptoms appeared between 7 months and 2 years after initiating dupilumab, with inflammation improving upon discontinuation in most cases and recurring upon reintroduction in one patient [9]. Therefore, the authors suggested a possible role of dupilumab in inducing intraocular inflammation, and such findings as a potential ocular adverse effect of the drug [9]. Mehta and colleagues described a case of a patient affected by atopic dermatitis that developed limbal stem cell deficiency and extensive symblepharon after prolonged dupilumab therapy [5]. The symptoms, which began a year into treatment, worsened over time, leading to vision impairment and gaze restriction [51]. Discontinuation of dupilumab resulted in partial improvement, but irreversible ocular damage remained [51]. Treatment included autologous serum eye drops, fluorometholone, and planned immunomodulators like cyclosporine [51]. The authors speculated the possible role of goblet cell loss and chronic inflammation on continuous limbal stem cell stress resulting in the disease manifestation [51] (Table 1).

## 5. Current Challenges and Future Directions

Despite growing awareness of dupilumab-associated ocular complications, significant challenges remain in understanding, predicting, and managing these adverse events. While a range of treatment strategies exists for managing dupilumab-associated ocular complications, there remains a lack of standardized guidelines, and therapeutic decisions are often empirical. Mild cases of conjunctivitis and keratitis typically respond well to conservative measures such as preservative-free artificial tears, trehalose, or antihistamine drops [5]. In contrast, moderate-to-severe cases may require escalation to topical corticosteroids (e.g., fluorometholone, loteprednol) or calcineurin inhibitors (e.g., tacrolimus, cyclosporine), which offer stronger anti-inflammatory effects with a reduced risk of systemic immunosuppression [5]. For refractory cases or those with recurrent inflammation, systemic agents such as oral doxycycline have shown benefit due to their dual anti-inflammatory and antimicrobial properties [5]. In rare, sight-threatening presentations—such as cicatricial conjunctivitis or corneal ulcers—temporary discontinuation or dose reduction of dupilumab has been effective in some reports [5]. Comparative evidence remains limited, but integrating treatment decisions with severity grading, patient comorbidities, and response to prior interventions could provide a more structured framework for individualized care. New mechanistic hypotheses and clinical observations have emerged, but they have yet to translate into standardized diagnostic criteria or treatment protocols. The following areas represent key directions for future research and clinical development. Although inhibition of IL-4 and IL-13 has been linked to goblet cell depletion and mucin deficiency, the full pathophysiological cascade remains elusive [15]. It is unclear why some patients develop mild, transient conjunctivitis while others experience chronic or cicatricial disease. The current literature suggest associations between pre-existing atopic ocular disease, elevated serum IgE, peripheral eosinophilia, and the risk of developing dupilumab-associated conjunctivitis [1]. However, no validated predictive model exists. A major challenge is the heterogeneity of clinical presentations and inconsistent baseline ophthalmologic screening in dupilumab trials. Prospective cohort studies with integrated biomarker analysis (e.g., tear cytokine profiles, conjunctival cell populations) could identify reliable predictors for ocular toxicity. Another key gap is the absence of a standardized classification system to stratify dupilumab-associated ocular side effects. The spectrum ranges from simple conjunctivitis to severe cicatricial forms and anterior uveitis, yet they are often grouped together under “ocular complications” in trials and reports. Differentiating phenotypes based on clinical features, duration, histopathologic findings, and response to therapy could facilitate tailored treatment and prognostication. Most of the current management approaches are empirical and based on small case series or expert opinion. However, there are no randomized controlled trials or consensus algorithms to guide therapy. Additionally, the role of prophylactic ophthalmic treatment at dupilumab initiation remains unexplored. Collaborative efforts are needed to develop evidence-based guidelines and validate treatment algorithms across specialties. Emerging evidence suggests a potential role of Demodex mites and Malassezia species in triggering periocular inflammation in dupilumab-treated patients [16]. These observations raise the possibility that microbial imbalance or unmasking of coexisting dermatoses (e.g., seborrheic dermatitis, rosacea) may modulate ocular outcomes. Future studies incorporating skin and ocular microbiome profiling, as well as patch testing for contact dermatitis, could help disentangle these overlapping entities. The long-term impact of dupilumab on ocular structures, particularly in patients with chronic inflammation or recurrent flares, remains unclear. There are limited data on whether early intervention alters disease trajectory or prevents vision-threatening complications. Additionally, the burden of ocular symptoms on patient-reported quality of life has not been adequately quantified. Inclusion of ophthalmic assessments and vision-related quality of life metrics in long-term registries and real-world studies is strongly recommended. Given that systemic inhibition of IL-4/IL-13 may inadvertently affect ocular tissues, future research may explore targeted delivery mechanisms or ocular-sparing biologics. The development of topical dupilumab analogs or conjunctiva-specific formulations could mitigate adverse effects while preserving therapeutic efficacy for skin or airway disease. As the use of dupilumab expands across dermatology, pulmonology, and allergy-immunology, the need for a multidisciplinary approach to ocular adverse effects is more pressing than ever. Addressing the current challenges will require collaborative efforts across basic science, clinical research, and real-world data collection. A deeper understanding of underlying mechanisms, risk stratification tools, and standardized treatment protocols will not only improve patient outcomes but also inform the safe use of future biologics targeting type 2 inflammation.

## 6. Conclusions

Dupilumab has revolutionized the management of atopic diseases, providing significant therapeutic benefits. However, its use has been associated with a wide range of ocular complications. Given the expanding use of dupilumab across multiple atopic conditions, and the potential impact of ocular side effects of patients’ quality of life and adherence to the therapy, an increased awareness among dermatologists, allergists, and ophthalmologists is essential for an early recognition and a prompt treatment. Future research should focus on elucidating the precise pathophysiology of these adverse effects, identifying predictive risk factors, and developing targeted therapeutic approaches to mitigate ocular inflammation without compromising the systemic benefits of dupilumab therapy.

## Figures and Tables

**Table 1:** List of clinical trials included in the manuscript.

CT Number	Status	Type	Phase	Enrollment	Ocular Safety Outcome
NCT02277743	Completed	RCT, Multicenter	Phase 3	1379	Conjunctivitis reported in 14% of dupilumab-treated AD patients
NCT03054428	Completed	RCT, Multicenter	Phase 3	740	Higher conjunctivitis incidence in dupilumab vs. placebo in AD
NCT02528214	Completed	RCT, Multicenter	Phase 3	210	Conjunctivitis occurred in 7% of dupilumab-treated asthma patients
NCT02407756	Completed	RCT, Multicenter	Phase 3	276	No increase in ocular AEs in CRSwNP patients
NCT03633617	Completed	RCT, Multicenter	Phase 2	67	No significant conjunctivitis events reported in EoE

## Abbreviations

AD	Atopic Dermatitis
CT	Clinical Trial
DAMD	Dupilumab-Associated Mucin Deficiency
DAC	Dupilumab-Associated Conjunctivitis
EMA	European Medicines Agency
EASI	Eczema Area and Severity Index
FDA	Food and Drug Administration
IL	Interleukin
MUC5AC	Mucin 5AC
TBUT	Tear Film Break-Up Time
Th2	T-helper 2 cells
RCT	Randomized Controlled Trial
